# Predictors of Empathy in Dental Education: Insights from a Cross-Sectional Measurement Using the Jefferson Scale

**DOI:** 10.3290/j.ohpd.c_2537

**Published:** 2026-03-04

**Authors:** Faisal Ali Bin Abbooud AlQhtani, Meer Zakirulla, Zuhair Motlak Alkahtani, Bandar Yahya Alshehri, Malaz M. Mustafa, Sana Mofleh Alshahrani

**Affiliations:** a Faisal Ali Bin Abbooud AlQhtani Assistant Professor, Department of Pediatric Dentistry and Orthodontic Sciences, College of Dentistry, King Khalid University, Abha, Saudi Arabia. Conceptualization, methodology, investigation formal analysis, prepared original draft, proofread the manuscript, consulted on and performed statistical evaluation, contributed substantially to discussion.; b Meer Zakirulla Assistant Professor, Department of Pediatric Dentistry and Orthodontic Sciences, College of Dentistry, King Khalid University, Abha, Saudi Arabia. Conceptualization, methodology, investigation, formal analysis, project administration, proofread the manuscript, consulted on and performed statistical evaluation, contributed substantially to discussion.; c Zuhair Motlak Alkahtani Associate Professor, Department of Pediatric Dentistry and Orthodontic Sciences, College of Dentistry, King Khalid University, Abha, Saudi Arabia. Conceptualization, methodology, investigation formal analysis, writing-original draft preparation, reviewed and edited manuscript, supervision, project administration.; d Bandar Yahya Alshehri Assistant Professor, Department of Pediatric Dentistry and Orthodontic Sciences, College of Dentistry, King Khalid University, Abha, Saudi Arabia. Reviewed and edited manuscript, supervision, project administration, proofread the manuscript, consulted on and performed statistical evaluation, contributed substantially to discussion.; e Malaz M. Mustafa Assistant Professor, Department of Pediatric Dentistry and Orthodontic Sciences, College of Dentistry, King Khalid University, Abha, Saudi Arabia. Conceptualization, methodology, investigation formal analysis, wrote original draft, proofread the manuscript, consulted on and performed statistical evaluation, contributed substantially to discussion.; f Sana Mofleh Alshahrani Assistant Professor, Department of Pediatric Dentistry and Orthodontic Sciences, College of Dentistry, King Khalid University, Abha, Saudi Arabia. Conceptualization, methodology, investigation formal analysis, wrote original draft, reviewed and edited manuscript. All authors have read and agreed to the published version of the manuscript.

**Keywords:** cross-sectional study, dental student, empathy, Jefferson Scale, Saudi Arabia.

## Abstract

**Purpose:**

Empathy is a vital attribute for effective patient care in dentistry, yet studies indicate a concerning decline in empathy levels during academic training. This research addresses the influence of gender and academic progression on dental students’ empathy levels. This study aimed to investigate variations in empathy levels among dental students at King Khalid University and to examine the associations between gender, academic year, and empathy levels.

**Materials and Methods:**

A cross-sectional study was conducted between January and May 2024. The study included 204 dental students (122 males, 82 females) with a mean age of 21.18 (± 2.43) years, representing an 81.6% response rate. Empathy was assessed using the validated Jefferson Scale of Empathy-Health Profession Students (JSE-HPS). Statistical analysis was performed using independent samples t-tests and one-way ANOVA to compare the mean scores.

**Results:**

A statistically significant difference in overall empathy was observed between genders (p = 0.002), with female students (mean = 108.34, SD = 14.23) reporting higher scores than male students (mean = 101.20, SD = 13.30). Additionally, empathy scores varied statistically significantly across academic years (p = 0.001), indicating a general decline from the first year (mean = 108.37, SD = 13.97) to the fifth year (mean = 98.33, SD = 15.12). Females outperformed males on all three subscales: perspective taking, compassionate care, and walking in patients’ shoes. The trajectories of the subscales exhibited variability, with “compassionate care” peaking in the first year, while “perspective taking reached” its highest point in the third year.

**Conclusions:**

Empathy in dental students is statistically significantly influenced by both sex and academic progression. These findings highlight the critical need for targeted educational strategies to foster and sustain empathy throughout dental training, thereby ensuring the development of patient-centered practitioners.

Empathy is an indispensable attribute for healthcare professionals, particularly within the demanding field of dentistry, as it underpins effective patient care and the establishment of strong patient-provider relationships.^12,21^ This critical trait enables practitioners to accurately understand and respond to patients’ concerns, fostering trust, improving communication, and enhancing diagnostic accuracy and treatment adherence.^1,18,24^ For dental students, developing strong empathetic capabilities is crucial for navigating complex clinical scenarios and ensuring positive patient outcomes.^2,12,19^ The American Dental Education Association highlights the ability to demonstrate empathy as a paramount clinical competency for new dentists, second only to ethical conduct.^2^

However, research has consistently indicated a worrying trend: empathy levels among healthcare students, including those in dentistry, often decline as they progress through their academic and clinical training.^19,22^ This decline is often attributed to various stressors inherent in medical and dental curricula, such as intense clinical experience, rigorous academic requirements, and an overwhelming focus on technical competence.^2^ Dental professionals, from the earliest stages of their education, face a unique set of pressures, balancing demanding didactic coursework, extensive laboratory work, and direct patient care under supervision.^13^

These factors collectively contribute to a substantial clinical workload and considerable academic stress, increasing the risk of psychological distress.^10^ Such intense and prolonged exposure to stressors can lead to burnout and compassion fatigue, wherein individuals experience emotional exhaustion, depersonalization, and a reduced sense of personal accomplishment.^3,7^ Burnout has been identified as a statistically significant predictor of negative outcomes, affecting students’ academic development and overall well-being.^4^ While some studies suggest that empathy may stabilize or even increase in later years, potentially due to ethics training and increased patient interaction,^2^ the general trajectory points to a vulnerability of these crucial humanistic traits amid the rigors of professional formation.^2^

Understanding how factors such as sex and academic year impact empathy levels among dental students is essential for designing targeted educational interventions.^8,21,26^ The extant literature reveals a complex relationship between empathy and the demanding academic and clinical environments encountered by dental students.^26^ Although empathy is recognized as a protective factor against burnout in medical professionals, its trajectory throughout undergraduate dental education remains debatable. Some studies indicate a decline in empathy during clinical training,^2^ while others suggest that empathy levels may stabilize or even increase in later years, potentially owing to enhanced ethics training and increased patient interaction.^19^ For instance, some research highlights a possible increase in empathy during the fifth and final years of dental education, likely influenced by direct patient-care experiences.^2^

Despite numerous studies on empathy in health professions education, findings on its trajectory and gender differences remain inconsistent and are scarce from Saudi dental schools, particularly at King Khalid University. This study addresses a critical gap by investigating empathy patterns among dental students at King Khalid University using a cross-sectional design to capture prevailing trends.^12^ By examining the relationships between gender, academic progression, and empathy levels, this research makes an important contribution to the literature on professional identity formation and well-being in dental education. The findings will inform the development of targeted interventions and curriculum adjustments designed to cultivate and sustain empathy, ensuring that future dental practitioners are not only technically proficient, but also deeply committed to patient-centered care.

## MATERIAL AND METHODS

This cross-sectional study was conducted between January 2024 and May 2024. The study population consisted of students enrolled in the College of Dentistry, King Khalid University, Saudi Arabia. Prior to the commencement of the study, formal ethical approval was secured from the Scientific Research Committee of the College of Dentistry at King Khalid University [IRB/KKU/COD/ETH/2023-24/027]. A target sample of 250 participants was recruited using a two-stage sampling strategy to ensure representation across all the academic years. Of the 250 students approached, 215 agreed to participate (86% agreement rate), but only 204 satisfied the inclusion criteria, yielding a final response rate of 81.6%. The target sample size of 250 students was determined a priori, based on power analyses designed to detect medium effect sizes in empathy scores between sexes and across academic years (Cohen’s d = 0.5; f = 0.25). Statistical significance was set at α = 0.05 with a statistical power of 0.80. This sample size also accounted for anticipated non-response and the exclusion of incomplete questionnaires. The planned recruitment was expected to yield a minimum of 200 analyzable responses, with sufficient representation across academic years to ensure precise estimation of mean JSE-HPS scores. The students were selected using systematic sampling with predetermined sampling intervals. The first student was chosen randomly within the interval and the class register was used to maintain the sampling order.

### Inclusion and Exclusion Criteria

#### Inclusion criteria

Currently enrolled undergraduate dental students
Voluntary participation with informed consent
Completion of all required questionnaires

#### Exclusion criteria

Students with pre-existing medical or psychiatric conditions that may affect empathy assessment
Exchange or visiting students not part of the regular curriculum
Students who withdrew consent during the study period

### Data Collection

All potential participants were provided with detailed information regarding the aims and objectives of the study. It was emphasized that participation was voluntary, and that all data would be anonymized and treated confidentially. Written informed consent was obtained from all the participants. The survey was conducted using a self-administered standardized questionnaire distributed to the participants in English. A timetable for each year was obtained from the Administration Unit, and a time schedule was drawn for data collection from each batch of students to ensure that all students were approached and had sufficient time to complete the questionnaire.

Data were gathered using a self-administered, structured questionnaire distributed to students in their classrooms or during mandatory academic sessions to maximize the response rate. A period of 10–15 minutes was allotted for completion. Researchers remained present during the data collection to clarify any ambiguities.

### Research Instruments 

The data collection instrument was a questionnaire divided into two parts.

1. Demographic and background Information: This section collected data on participant age, sex, and year of study.

2. Empathy was measured using the Jefferson Scale of Empathy-Health Profession Student (JSE-HPS) version. The scale is categorized into three subscales:

*Perspective taking* This is the cognitive dimension of empathy. It refers to the intellectual ability to understand a patient’s pain, suffering, and emotional state without necessarily feeling those emotions oneself. It involves “theory of mind”—the deliberate mental process of accurately inferring another person’s thoughts and motives.
*Compassionate care *This is the affective or emotional dimension of empathy. It measures the degree to which a student values the emotional connection in the patient-provider relationship and rejects the notion that emotions are irrelevant to treatment.
*Walking in patient’s shoes *This specific subscale measures the active capacity or perceived ease of empathizing with a patient. While similar to perspective taking, it specifically isolates the difficulty a provider feels when trying to view the world from the patient’s viewpoint.

This is a validated 20-item instrument in which responses are captured on a 7-point Likert scale (1 = strongly disagree to 7 =  strongly agree). Positively and negatively worded items were included, and the latter were reverse-scored during the analysis. The total empathy score (range: 20–140) was calculated by summing the item scores, with higher scores signifying greater levels of empathy; by subscale, these comprise perspective-taking with 10 positively scored items, compassionate care with eight reverse-scored items, and walking in the patient’s shoes with two reverse-scored items. Higher scores indicate greater empathy adoption by healthcare workers toward patients. The validity and reliability of this questionnaire have been confirmed in numerous studies.^1,5,20,27,30,31^ The JSE-HPS has high reliability, as verified by the α coefficient of 0.89 for dental students.

### Data Analysis 

All collected data were compiled in an Excel spreadsheet, validated, and exported to SPSS for statistical analysis. Questionnaires with significant missing data (i.e., >20% of unanswered items) were excluded from the final analysis. Descriptive statistics were used to summarize the data. Categorical variables (e.g., sex, year of study) were presented as frequencies (n) and percentages (%), while continuous variables (e.g., age and empathy scores) were reported as means and standard deviations (SD). Inferential statistics were used to compare and explore the associations. The independent samples t-test was used to compare mean scores between two independent groups (e.g., by gender), and one-way ANOVA was used for comparisons among three or more groups (e.g., across academic years). Pearson’s correlation coefficient was computed to assess the linear relationship between empathy scores and the other continuous variables. For all statistical tests, p < 0.05 was adopted as the threshold for statistical significance.

## RESULTS

**Fig 1:**
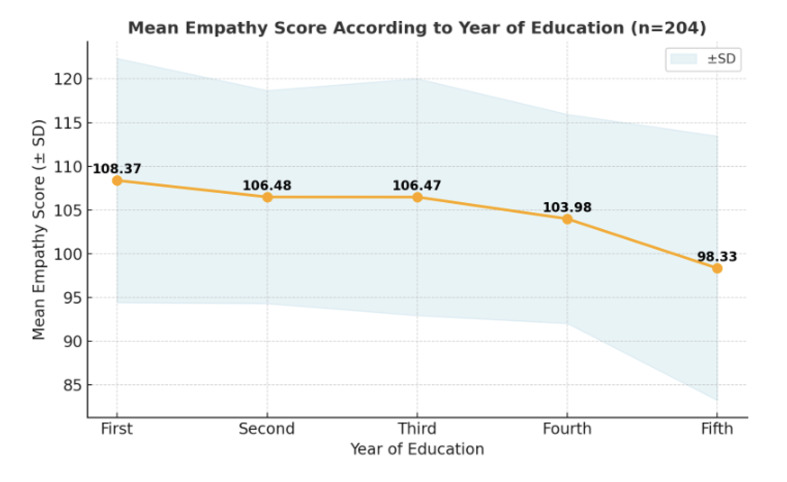
Line graph showing the mean empathy score according to the year of education. The shaded area represents the standard deviation (± SD), indicating the variability in empathy scores within each year.

The study included 204 participants with an age range of 17 to 32 years and a mean (SD) of 21.18 years (2.43). Of the participants, 122 (59.8%) were males and 82 (40.2%) were females. A statistically significant difference was observed in empathy scores between the sexes (p = 0.002). Females (mean 108.34 ± 14.23) reported higher empathy scores than males (mean 101.20 ± 13.30), representing a moderate effect size (Cohen’s d = 0.53) (Table 1). A statistically significant difference was also found across the years of education (p = 0.001). Empathy scores were highest among first-year students (mean 108.37 ± 13.97) and showed a general decline, with fifth-year students (mean 98.33 ± 15.12) and fourth-year students (mean 103.98 ± 11.96) reporting the lowest scores. The mean empathy score gradually declined from the first to the fifth year, with the highest mean score in the first year (108.37) and the lowest in the fifth year (98.33) (Fig 1).

**Table 1:** Mean empathy score of the students categorized according to their genders and year of education (n = 204)

	Frequency (%)	Empathy score (mean ± SD)	p-value
**Gender**			0.002*
Male	122 (59.8%)	101.20 ± 13.30	
Female	82 (40.2%)	108.34 ± 14.23	
**Year of education**			0.001**
First	32 (15.7%)	108.37 ± 13.97	
Second	51 (25.0%)	106.48 ± 12.19	
Third	45 (22.1%)	106.47 ± 13.55	
Fourth	35 (17.1%)	103.98 ± 11.96	
Fifth	41 (20.1%)	98.33 ± 15.12	
*p<0.05, **p<0.01.			

### Subscale Analysis 

**Fig 2:**
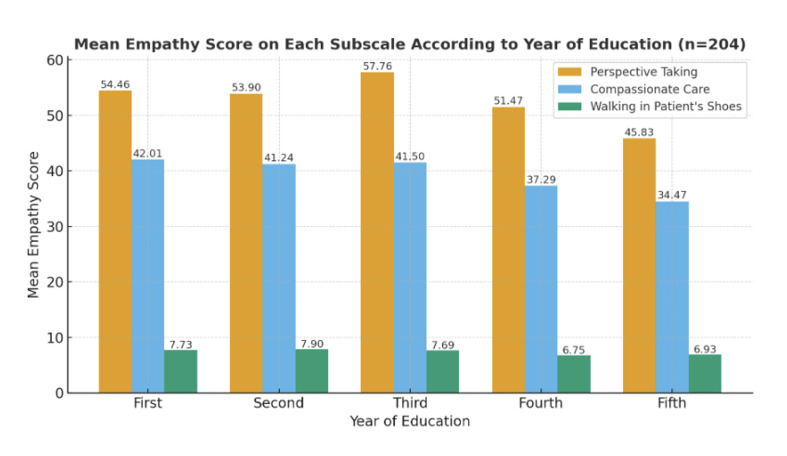
Mean empathy score on each subscale categorized according to the genders and year of education of the students (n = 204).

Females showed higher empathy scores in subscales, analyzing 204 participants (122 males, 82 females). The average “perspective taking” score was 61.75 ± 10.80 for females and 52.82 ± 5.30 for males. For “compassionate care,” scores were 37.90 ± 4.25 for females and 36.80 ± 5.90 for males. In the “walking in patient’s shoes” subscale, females scored 9.07 ± 2.75, while males scored 7.82 ± 3.10 (Table 2). Across the years of education, “perspective taking” scores peaked in the third year (mean 57.76 ± 7.10) and were lowest in the fifth year (mean 45.83 ± 7.20). “Compassionate care” scores were highest among first-year students (mean 42.01 ± 6.10) and lowest in the fifth year (mean 34.47 ± 5.20). The “walking in patient’s shoes” subscale showed less variation, with a peak in the second year (mean 7.90 ± 3.10) and the lowest scores in the fourth year (mean 6.75 ± 2.80) (Fig 2).

**Table 2:** Mean empathy score on each subscale categorized according to the genders and year of education of the students (n = 204)

	Perspective taking	Compassionate care	Walking in patient’s shoes
**Gender**			
Male (n = 122)	52.82 ± 5.30	36.80 ± 5.90	7.82 ± 3.10
Female (n = 82)	61.75 ± 10.80	37.90 ± 4.25	9.07 ± 2.75
**Year of education**			
First (n = 32)	54.46 ± 9.50	42.01 ± 6.10	7.73 ± 3.20
Second (n = 51)	53.90 ± 10.20	41.24 ± 7.30	7.90 ± 3.10
Third (n = 45)	57.76 ± 7.10	41.50 ± 5.80	7.69 ± 2.90
Fourth (n = 35)	51.47 ± 9.90	37.29 ± 7.40	6.75 ± 2.80
Fifth (n = 41)	45.83 ± 7.20	34.47 ± 5.20	6.93 ± 3.60


## DISCUSSION

This study examined empathy in dental students at King Khalid University. It found key differences based on sex and academic year. Female students showed higher empathy scores than did male students. Empathy decreased as students advanced. First-year students had the highest empathy and fifth-year students the lowest. These findings highlight areas for improvement in dental education. These results underscore the critical areas for intervention and further inquiry into dental education.^9^

### Empathy Decline Across Academic Years 

Our findings on declining empathy align with studies reporting similar trends in healthcare education, including dentistry.^1,2,6^ This phenomenon is often attributed to the inherent stressors of demanding curricula, intense clinical experiences, and overwhelming focus on technical competence, which may reduce students’ empathetic responses. While some studies suggest potential stabilization or even an increase in empathy during later clinical years due to increased patient interaction and ethics training,^2,14^ our cross-sectional data indicate a continuous downward trajectory until the final year, with the lowest scores among fourth- and fifth-year students. Among the studies cited, most reports in dental and allied health education document lower empathy in senior (final) years, consistent with the downward trajectory observed in our cross‑sectional sample.^1,26^ A smaller group of studies, predominantly in medicine and pharmacy, describe stable or increasing empathy scores in later clinical years, often in the context of curricula emphasizing early patient contact and structured communication or ethics training.^22^ These discrepancies likely reflect differences in student populations, cultural context, curricular design, and measurement tools, as well as cross‑sectional vs longitudinal approaches, and thus do not necessarily contradict our findings but rather highlight the contextual sensitivity of empathy trajectories in health‑professional education.^1,22,26^ Table 3 summarizes the nine studies that specifically examined empathy trajectories across academic progression, highlighting substantial heterogeneity in reported patterns according to discipline, study design, and curricular context. Conversely, it is important to acknowledge that not all studies report a decline, with some indicating stable or increasing empathy levels in medical education, suggesting the influence of diverse curricular models and learning environments.^22^ For instance, a systematic review of pharmacy students found varied trends in empathy levels, sometimes showing stability or even an increase.^22^ However, other research, particularly concerning radiography students, has identified a statistically significant drop in empathy during the third year of education, correlating with heightened burnout levels.^1^ This consistent decline across various health professions underscores a broader challenge within healthcare education, indicating that curriculum and clinical demands may inadvertently suppress empathetic responses rather than foster them.^2^ This reduction in empathy, as observed in our cohort and corroborated by other studies, may stem from the substantial academic pressures and clinical demands placed upon students, which could lead to emotional exhaustion and detachment.^26,27^ The observed decline may also be exacerbated by limited patient interaction in the early stages and lack of specific feedback on empathetic communication, as reported in studies involving pharmacy students.^27^ The phenomenon of declining empathy among health professional students is well documented, with some studies indicating that this reduction is most pronounced during the preclinical years before stabilizing or potentially rebounding in the later clinical phases.^27^

**Table 3:** Summary of studies examining empathy trajectories across academic years in undergraduate students

Authors (year)	Discipline / cohort	Study design	Empathy trajectory reported	Key notes / relevance to present study
Abuzaid et al (2025)^1^	Radiography	Cross-sectional	Decline in advanced years	Non-dental cohort; decline associated with increased clinical stress and burnout
Ameh et al (2025)^2^	Medicine	Longitudinal	Stable or increasing empathy	Same cohort followed; later-year curriculum included communication skills
Baig et al (2025)^5^	Medicine	Cross-sectional	Lower empathy in senior years	Supports decline hypothesis but outside dental education
Chen et al (2025)^6^	Medicine	Cross-sectional	Decline with academic progression	Clinical workload cited as contributing factor
Díaz-Narváez et al (2025)^9^	Dentistry (Latin America)	Multicenter cross-sectional	Challenges “empathy decline” narrative	Explicitly questions linear decline; strong regional influence
Mirani et al (2025)^17^	Mixed health professions	Cross-sectional	Reduced empathy in later years	Heterogeneous sample; limits direct comparability
Khademalhosseini et al (2025)^14^	Medicine	Longitudinal	Empathy maintained or increased	Ethics and medical humanities embedded in senior curriculum
Walker et al (2025)^27^	Pharmacy	Longitudinal	Stable or increasing empathy	Structured professional identity and communication training
Wang et al (2025)^28^	Physiotherapy / health sciences	Cross-sectional	Lower empathy in advanced years	Conducted in a culturally distinct educational setting


### Gender Differences in Empathy 

The higher empathy scores among female students are consistent with numerous studies conducted across various healthcare disciplines, including dentistry.^14,22^ This gender difference in empathy is a consistent finding, often linked to the distinct ways in which individuals process emotional information and relate to others. However, the research findings are not completely consistent, as some studies reveal no notable gender disparities, or even higher empathy scores for men in particular situations,^1,2^ which emphasizes empathy’s complexity and the possible impact of cultural and educational environments. Nevertheless, this study’s consistent observation of higher empathy in females aligns with prevailing literature, which often attributes such disparities to inherent psychological and sociological factors influencing emotional expression and relational engagement.^21^ For instance, studies, such as those involving female dental students in Nigeria, consistently demonstrate higher, though not always statistically significant, empathy scores among females compared to their male counterparts, echoing a prevailing pattern observed across global healthcare education.^2^ This consistent gender difference may be influenced by societal roles and expectations, which often encourage females to express emotions more openly and cultivate interpersonal sensitivity, thereby fostering greater empathic capacity.^21^ Furthermore, certain cultural and educational factors could contribute to these observed disparities, emphasizing the importance of considering environmental influences on empathy development. These patterns may partially reflect cultural and environmental characteristics of Saudi dental education. In this context, strong religious and societal norms emphasize compassion and helping others, which may contribute to the relatively high empathy scores observed among first year students upon entering the program. However, as students progress through a traditionally teacher-centered, examination-intensive curriculum with increasing clinical responsibility, academic stress, and performance pressure may erode these initially highly empathetic attitudes, contributing to the downward trajectory observed across academic years. The consistently higher empathy scores among female students could also be interpreted within prevailing gender norms in Saudi society, where women may be more encouraged to express emotions and engage in caregiving roles, potentially facilitating greater perspective taking and compassionate care in clinical interactions. Because these cultural and environmental influences are specific to our institutional and national context, caution is needed when generalizing these findings to dental schools operating in different sociocultural settings. For example, a study comparing empathy levels among health professions students found that female physiotherapy students consistently scored higher than males, even after targeted educational interventions.^25^ This consistent finding across diverse contexts underscores the need for targeted interventions within dental curricula to cultivate and sustain empathy among all students, particularly male students, throughout their academic journey.^9,21^

### Subscale Analysis Insights 

The Jefferson Scale of Empathy, which was utilized in this study, is a well-established and validated instrument for assessing empathy in healthcare students. The subscale analysis revealed specific patterns, such as “perspective taking” peaking in the third year and “compassionate care” being highest in the first year, suggesting that different facets of empathy may be affected differently by the educational environment. This differential impact on subscales indicates that empathy is a multidimensional construct, with various components responding distinctly to academic progression and clinical exposure.^29^ It is crucial for educational institutions to recognize these nuanced changes to develop targeted interventions that bolster specific empathetic capacities throughout the curriculum rather than solely focusing on a general decline.^1^ This phenomenon of declining empathy during the intense clinical years is well-documented in medical and pharmacy students, often coinciding with heightened academic pressures and exposure to patient suffering without adequate emotional support mechanisms.^19,25,27^ Moreover, this decline in empathy has been noted to be more pronounced in certain dimensions, such as “empathic concern” and “personal distress,” as students navigate the complexities of clinical practice.^26^ Furthermore, while some studies indicate that female students consistently exhibit higher empathy scores, particularly in the compassionate care subscale, other research suggests no statistically significant gender differences, or even higher male empathy in specific contexts.^1,9,12^ Such discrepancies in findings necessitate further investigation into the cultural, social, and programmatic factors that might influence the manifestation of empathy across genders.^6,26^ Affective (emotional) empathy is the capacity to share or “catch” another person’s emotional state, such as feeling sad when a patient is distressed and it relies strongly on emotional contagion and empathic concern, helping to create warmth and emotional closeness in clinician–patient relationships. Cognitive empathy is the ability to accurately infer and understand another person’s internal state, perspective, and motives, often described as “perspective taking.” It involves deliberate mental processes (theory of mind), such as adjusting explanations to a patient’s level of understanding or anticipating how a treatment plan might be perceived. The impact of academic efficacy on both affective and cognitive empathy further complicates this understanding, as it can influence empathy levels across different study years and genders.^26^ Therefore, a comprehensive approach to empathy education should consider these multifaceted interactions to mitigate the observed decline and promote sustained empathetic engagement among dental students.^2,26^

### Educational Implications 

Understanding these dynamics is vital for developing targeted educational interventions that can sustain and cultivate empathy throughout a dental student’s academic journey, particularly when they encounter increasing clinical demands and academic pressures.^28^ For example, integrating humanities programs or dedicated communication skills training throughout the curriculum—not only as isolated courses—could help mitigate the observed decline in empathy, particularly during the later clinical years when academic and clinical stressors intensify. These interventions should be intentionally longitudinal, with repeated opportunities for reflective writing, role‑play, and simulated patient encounters that explicitly assess and give feedback on empathic behaviors rather than focusing solely on technical competence. Furthermore, given the documented correlation between empathy and burnout, with emotional exhaustion and depersonalization negatively impacting empathy, it is imperative to integrate resilience‑building strategies and stress management techniques into dental curricula, such as mindfulness‑based stress reduction, peer‑support groups, and structured debriefing sessions after emotionally demanding clinical experiences.^16^ In addition, because females consistently demonstrated higher empathy scores across all JSE‑HPS subscales in this study, whereas males showed comparatively lower scores, targeted educational approaches may be needed, for example: small‑group communication workshops and mentoring programs that explicitly engage male students in reflective discussion of patient perspectives and professional identity formation, while simultaneously reinforcing and sustaining empathy among female students to prevent erosion over time. This proactive and gender‑sensitive approach can foster a more empathetic and resilient generation of dental professionals, ultimately benefiting both practitioners and patients.^26^

The broader implications of these findings for dental education are significant. Empathy is the cornerstone of effective patient care, fostering trust, improving communication, and enhancing treatment adherence and patient satisfaction. The observed decline in empathy among dental students, particularly as they approach graduation, raises concerns about the preparedness of future practitioners to meet the humanistic demands of their professions. These results emphasize the urgent need for targeted educational strategies that both build and preserve empathy in dental students by focusing on the most vulnerable groups (male and senior year students) and the most affected empathy dimensions (especially compassionate care and perspective taking).^15,16,19^ Such strategies should be carefully aligned with academic year and clinical load, using early year interventions (e.g., introductory communication skills and humanities based activities) to cultivate high baseline empathy, and intensified, clinically integrated activities in later years (e.g., reflective case discussions, patient narrative sessions, and stress management support) to sustain empathy under increasing curricular and clinical pressures.^23,28,31^ Integrating patient-centered care and ethical considerations explicitly and consistently throughout the curriculum, rather than in isolation, could be crucial in mitigating this decline.

### Limitations 

Despite these important insights, this study had several limitations. The cross-sectional design limits our ability to establish causal relationships or track individual empathy trajectories over time. The reliance on a self-reported instrument, the Jefferson Scale of Empathy, may be subject to social desirability bias, in which participants might respond in a manner that they perceive as favorable. Furthermore, the study was conducted within a single institution, limiting the generalizability of the findings to other dental schools or diverse cultural contexts.^2^ Additionally, while the study title and introduction discuss clinical workload and academic stress, these factors were not directly measured in the current study, preventing a detailed analysis of their specific impact on empathy levels.^15^

### Future Research Directions 

Future research should address these limitations by employing longitudinal study designs to track changes in empathy over time within cohorts of dental students, allowing a clearer understanding of the causal factors and individual variations. It is also imperative for subsequent studies to thoroughly investigate the specific impact of clinical workload and academic stress on empathy, ensuring that these factors are robustly measured, and their findings are explicitly presented and analyzed.^17^ Multi-institutional studies would enhance the generalizability of the findings, and the integration of qualitative methods could provide deeper insights into the lived experiences of dental students and the contextual factors influencing their empathy development. Finally, the development and rigorous evaluation of educational interventions aimed at fostering and maintaining empathy throughout dental training are critical for ensuring that future dental practitioners are not only technically proficient but also deeply attuned to the needs of their patients.^12^

## CONCLUSION

This study provides valuable insights into the dynamics of empathy among dental students at King Khalid University, highlighting statistiscally significant gender differences and a disquieting decline across academic years. These findings reinforce the critical need for dental education programs to prioritize the continuous cultivation and preservation of empathy. By understanding these predictors, we can inform the development of more effective curricula and supportive learning environments that ensure the emergence of dental professionals who are both clinically skilled and patient centered, thereby strengthening the foundation of effective healthcare delivery.

## ACKNOWLEDGEMENT

The authors extend their appreciation to the Deanship of Research and Graduate Studies at King Khalid University for funding this work through Large Research Project under grant number RGP2/526/46.

## APPENDIX

**Table A1:** Item-wise mapping of the Jefferson Scale of Empathy–Health Professions Student Version (JSE-HPS)

Item No.	Item Statement	Subscale
1	Empathy is a therapeutic skill without which a health care provider’s success is limited	Perspective-taking
2	Patients value a health care provider’s understanding of their feelings	Perspective-taking
3	Understanding patients’ and families’ emotional status is an important component of care	Perspective-taking
4	Patients feel better when providers understand their feelings	Perspective-taking
5	Empathy is an important factor in patients’ treatment	Perspective-taking
6	Providers should understand patients’ minds through non-verbal cues and body language	Perspective-taking
7	Understanding body language is as important as verbal communication	Perspective-taking
8	Providers should stand in their patients’ shoes	Perspective-taking
9	Providers should think like their patients to render better care	Perspective-taking
10	Emotion has no place in the treatment of medical illness	Compassionate care
11	Asking about patients’ personal lives is not helpful	Compassionate care
12	Understanding patients’ and families’ feelings does not influence outcomes	Compassionate care
13	Attentiveness to patients’ personal experiences does not influence outcomes	Compassionate care
14	Attention to patients’ emotions is not important in patient interview	Compassionate care
15	Patients’ illnesses can be cured only by targeted treatment; emotions do not matter	Compassionate care
16	A provider’s sense of humor contributes to better clinical outcomes	Perspective-taking
17	Providers should not be influenced by strong personal bonds with patients/families	Compassionate care
18	I do not enjoy reading non-medical literature or the arts	Compassionate care
19	It is difficult to see things from patients’ perspectives	Walking in the patient’s shoes
20	It is difficult for a provider to view things from patients’ perspectives	Walking in the patient’s shoes

